# Effects of In-Bed Cycle Exercise in Patients With Acute Stroke: A Randomized Controlled Trial

**DOI:** 10.1016/j.arrct.2020.100085

**Published:** 2020-09-28

**Authors:** Klas Sandberg, Marie Kleist, Magnus Wijkman, Paul Enthoven

**Affiliations:** a Department of Rehabilitation Vrinnevi Hospital, Norrköping, Sweden; b Department of Health, Medicine and Caring Sciences, Linköping University, Norrköping, Sweden; c Department of Internal Medicine, Vrinnevi Hospital, Norrköping, Sweden

**Keywords:** Exercise, Randomized controlled trial, Rehabilitation, Stroke, BI, Barthel Index, IQR, interquartile range, mRS, modified Rankin Scale, NIHSS, National Institutes of Health Stroke Scale, RPE, rating of perceived exertion, 6MWT, 6-minute walk test

## Abstract

**Objective:**

To investigate the effects of in-bed cycle exercise in addition to usual care in patients with acute stroke, National Institutes of Health Stroke Scale (NIHSS) 7-42, regarding walking ability, functional outcomes, and inpatient care days.

**Design:**

Randomized controlled trial.

**Setting:**

Hospital care.

**Participants:**

Patients (N=56) with stroke NIHSS 7-42 were recruited 24-48 hours after stroke onset from 2 stroke units in Sweden.

**Interventions:**

Both groups received usual care. The intervention group also received 20 minutes bed cycling 5 days per week with a maximum of 15 sessions.

**Main Outcome Measures:**

The primary outcome was median change in walking ability measured with the 6-minute walk test (6MWT). Secondary outcome measures included the median change in modified Rankin Scale (mRS), Barthel Index (BI) for activities of daily living, and inpatient care days. Measurements were performed at baseline, post intervention (3 weeks), and at 3-month follow-up.

**Results:**

There was no significant difference in change of walking ability (6MWT) from baseline to follow-up between the intervention and control groups (median, 105m [interquartile range [IQR, 220m] vs 30m [IQR, 118m], respectively, *P*=.147, *d*=0.401). There were no significant differences between groups regarding mRS, BI, or inpatient care days. Patients with less serious stroke (NIHSS 7-12) seemed to benefit from the intervention.

**Conclusion:**

Although this study may have been underpowered, patients with stroke NIHSS 7-42 did not benefit from in-bed cycle exercise in addition to usual care after acute stroke. A larger study is needed to confirm our results.

Development of poststroke rehabilitation is urgent. Personal suffering and high health care costs remain high. Despite the increased interest in early poststroke activities in recent years, detailed knowledge of exercise prescription is still lacking.^1^ We need a better understanding of what interventions and doses to use to optimize recovery from stroke. Previous studies using cycle ergometry in the subacute stage of stroke have shown beneficial effects on functional capacity, balance, and cardiovascular fitness and beneficial effects on walking ability in chronic stroke.^2^, ^3^, ^4^, ^5^, ^6^, ^7^ However, little is known of the effects of cycle ergometry in the acute stage of stroke.

Experimental studies provide a possible rationale to the effect and support early exercise to enhance spontaneous recovery. Synthesized findings from Austin et al^8^ showed that early (24-48h post stroke) initiation of moderate forced exercise (10m/min, 5-7d/wk for about 30min) reduced lesion volume and protected perilesional tissue against oxidative damage and inflammation.^8^ Angiogenesis is believed to be an important physiological process in restorative processes after stroke.^8^ Although initially believed to be a developmental phenomenon, nonpathologic angiogenesis is now understood to occur in adult animals in response to exercise.^9^ There is knowledge from animal studies in this early rehabilitation phase, but there are few human studies. In-bed cycling exercise is a nonpharmacologic way to possibly stimulate cerebral repair processes through aerobic exercise to reach higher functional outcomes and is one of few possible exercise interventions for patients with severe stroke in the acute phase. Increased cerebral blood flow velocities have been demonstrated during active and passive exercise.^10^^,^^11^ Chen et al also showed that passive in-bed cycling provides a hemodynamic response to a graded increase in cadence, with mean arterial pressure increasing by 7%.^12^ Feasibility has been proven during tests in intensive care, with no or a small effect on intracranial pressure after early in-bed cycling and passive exercise.^13^ Nevertheless, there is a lack of studies exploring clinical outcomes from in-bed exercise in the acute poststroke period.

The aim of the study was to investigate the effects of in-bed cycling exercise in addition to usual care in patients with acute stroke, National Institutes of Health Stroke Scale (NIHSS) 7-42, regarding walking ability, functional outcomes, and inpatient care days.

## Methods

This study was a dual-center, parallel, prospective randomized controlled trial (NCT04241952). The study was approved by the Regional Ethics Committee, Linköping, Sweden DNR 2015/358-31. The study was guided by the Consolidated Standards of Reporting Trials statement.

### Participants and setting

Patients were recruited consecutively from the stroke unit at Vrinnevi Hospital, Norrköping, and Höglandssjukhuset, Eksjö in Sweden, during November 2015 to November 2018.

#### Inclusion criteria

The subjects had to be at least 18 years old, but there was no upper age limit. All subjects had to have had a first stroke that was diagnosed by a physician prior to the request for inclusion. Subjects had to be considered able to perform aerobic exercise by the responsible physician and to understand spoken and written instructions. Their impairments had to correspond to stroke NIHSS 7-42.^14^

#### Exclusion criteria

Exclusion criteria were medical or neurologic diseases that could either be a risk or make the exercise program difficult to fulfill. This judgment was made by the treating physician. Patients treated with thrombolysis were also excluded.

### Procedures

Participants were recruited consecutively from the stroke units by the responsible physiotherapist. The participants received written and oral information about the study. Written informed consent was obtained from all participants. At the start of the study 24-48 hours after onset (baseline/preintervention) and prior to randomization, a 6-minute in-bed cycle test and other physical assessments (subsequently listed) were carried out in the stroke unit. The assessments were repeated after 3 weeks (post intervention) at the stroke unit or at discharge from the stroke unit and after 3 months (follow-up). The participants’ physiotherapist and study-responsible physiotherapist were responsible for randomization. Randomization was performed by shuffling concealed envelopes after which the treating physiotherapist randomly picked an envelope. The intervention started 24-48 hours after randomization. At follow-up, all participants were visited by a physiotherapist in their home or at the relevant community ward.

### Intervention

#### Usual care

Both groups received usual care and rehabilitation, including early out-of-bed mobilization and sitting exercise. If possible, standing and walking exercise were conducted. General advice about physical training and activity was given, and participants were encouraged to try to return to their previous activity level as soon as possible.

#### Aerobic exercise program

According to Saunders,^15^ there is sufficient evidence to incorporate cardiorespiratory and mixed training within poststroke rehabilitation programs to improve the speed and tolerance of walking. The American Heart Association recommends 20- to 60-minute sessions of aerobic exercise training 3-5 days per week after stroke.^16^ The intensity should be 50%-80% of the maximal heart rate (11-14 on the Borg rating of perceived exertion scale).^17^ After the acute setting, participants were discharged to the stroke unit within the first 24 hours. Baseline testing and randomization were conducted 24-48 hours after arrival to the stroke unit. The intervention group began exercise after randomization. The exercise period lasted for 3 weeks and included daily sessions 5 days per week, resulting in a maximum of 15 sessions. The exercise sessions were conducted in the wardroom and included 20 minutes of aerobic in-bed cycling. New participants were included consecutively and continuously. The exercise sessions were led by an experienced physiotherapist. The individual exercise intensity was adapted during each session by adjusting the load or the cycling speed so that the exercise goals were achieved. If the participants did not spontaneously reach the target intensity and exercise time, the bed cycle provided active support and the physiotherapist gave verbal encouragement. Attendance at exercise sessions was recorded in the exercise log.

Each 20-minute session was performed in bed in supine position with an electrical bed cycle.a Each participant was encouraged to cycle by himself or herself, but otherwise the cycle was able to run passively at 20 revolutions per minute. Each participant was given 2 fitness goals for each exercise session. The first goal was to reach 20 minutes of cycling, active or passive. The second goal was to reach an exertion level rating of perceived exertion^11^, ^12^, ^13^ that corresponded to ≥50% of the estimated maximum oxygen uptake and 60% of the maximum heart rate.^17^^,^^18^

#### Comparison

In this study the intervention bed cycle exercise (intervention group) was compared with usual care only (control group).

### Primary outcome measure

Walking ability is one of the most important functions to recover after stroke.^19^^,^^20^

Walking distance was measured with the 6-minute walk test (6MWT), which is a commonly used test for assessing walking ability after stroke.^21^ The primary outcome measure was median change in 6MWT from baseline to follow-up.

### Secondary outcome measures

Disability degree was measured with modified Rankin Scale (mRS),^22^^,^^23^ and activity of daily living was measured with the Barthel Index (BI).^24^ Inpatient care days were measured at the stroke unit. Secondary outcome measures were median changes in mRS and the BI from baseline to follow-up and inpatient care days.

### Statistical analysis

The sample size calculation was based on the primary outcome measure, 6MWT. Using a 2-tailed test with a type I error of 0.05 and a power of 80%, a clinically signiﬁcant difference between the intervention and control groups (mean improvement, 50±53m) for the 6MWT would be detected with a minimum sample of 20 subjects per group.^25^ Considering possible dropouts, the primary study goal was to include at least 100 participants. Statistical analyses were conducted using SPSS version 25.b The level of signiﬁcance was set at *P*<.05. Descriptive statistics were used to analyze demographic and clinical characteristics (table 1). Normally distributed continuous variables are presented as mean ± SD and nonnormally distributed variables as median and interquartile range (IQR). Categorical data are presented as numbers and percentages. Between-group differences were tested for statistical significance with the chi-square test, the Fisher exact test, the Mann Whitney *U* test, and the unpaired *t* test as appropriate. Cohen *d* effect sizes were reported based on the Mann-Whitney *U* test statistic. For the effect size calculations, a website was used: https://www.psychometrica.de/. The following interpretation for the magnitude of the effect size is suggested: no effect (0-0.1), small effect (0.2-0.4), intermediate effect (0.5-0.7), and large effect (≥0.8).^26^

**Table 1:** Baseline characteristics of patients stratified by intervention or control group (usual care)

Variables	Intervention Group (n=23)	Control Group (n=29)	*P* Value
Age (y)			.128∗
Mean ± SD	72.1±11.7	76.3±6.4	
Range	50-89	61-91	
Sex			.627†
Male, n (%)	8 (34.8)	12 (41.4)	
Female, n (%)	15 (65.2)	17 (58.6)	
Type of stroke			.020‡
Ischemic, n (%)	23 (100)	23 (79.3)	
Hemorrhagic, n (%)	0 (0)	6 (20.7)	
Side affected by symptoms			.984†
Right, n (%)	12 (52.2)	15 (51.7)	
Left, n (%)	10 (43.5)	13 (44.8)	
Unknown, n (%)	1 (4.3)	1 (3.4)	
NIHSS			
Mean ± SD	13.0±4.8	13.2±4.1	.845∗
Median (IQR)	12 (6)	12 (6)	.677§
Stroke onset to randomization (d)			.151∗
Mean ± SD	1.9±1.0	2.6±1.8	
Median (IQR)	2 (2)	1 (1)	

NOTE. There were no significant differences in patient characteristics at baseline between the intervention and control group except that the intervention group included none, while the control group included 6 subjects with hemorrhagic type of stroke.

∗ Unpaired *t* test.

† χ^2^ test.

‡ Fisher exact test.

§ Mann-Whitney *U* test.

## Results

**Fig 1:**
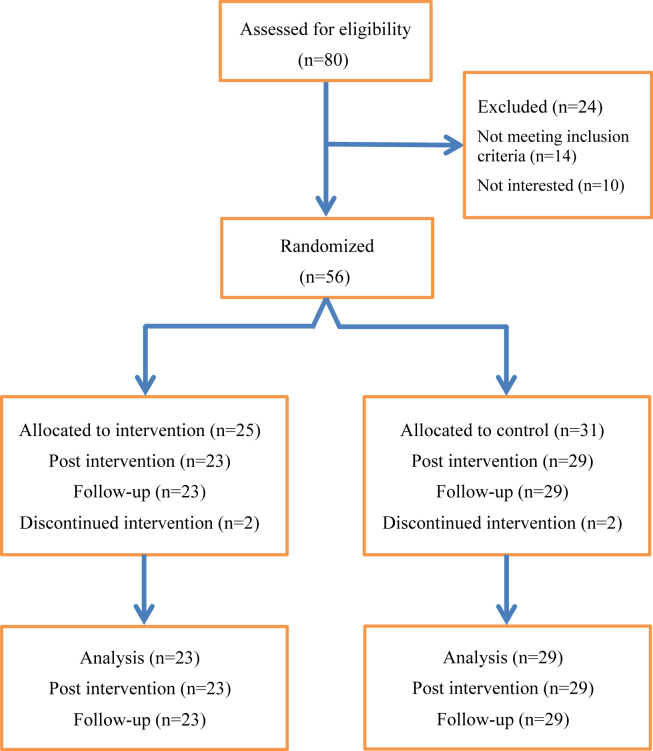
Flowchart of participants through each stage of the trial.

Between November 2015 and November 2018, a total of 80 participants were assessed for study eligibility. Recruitment stopped after 80 subjects were enrolled because of changes in routine in the clinics. Of these, 56 participants were included in the study. The reasons why participants declined participation are shown in fig 1. The participants were randomized early after stroke (median, 2d [IQR, 2d]) to either the intervention group (n=25) or the control group (n=31).

There were no significant differences in patient characteristics at baseline between the intervention and control groups except that the intervention group included none, while the control group included 6 subjects with hemorrhagic stroke (see table 1). No deaths occurred during the study or during follow-up. There were 4 dropouts, and 52 participants completed the study (see fig 1). Two participants, 1 from the intervention group and 1 from the control group, deteriorated during the care period for reasons not considered to be related to the study and were unable to follow up. Two participants, 1 from the intervention group and 1 from the control group, lack follow-up because of missed registration.

### Primary outcome measure

The change in walking distance (6MWT) from baseline to follow-up was numerically higher in the intervention group than in the control group, but the difference was not statistically significant (median, 105m [IQR, 220m] vs 30m [IQR, 118m], respectively; *P*=.147, *d*=0.401) (table 2).

**Table 2:** Primary and secondary outcome measures, comparisons between groups and over time

Measures	Intervention (n=23)	Control (n=29)	*P* Value∗	*d* Effect Size	Change From Baseline				Change From Post Intervention			
					Intervention (n=23)	Control (n=29)	*P* Value∗	*d* Effect Size	Intervention (n=23)	Control (n=29)	*P* Value	*d* Effect Size
	Median (IQR)	Median (IQR)			Median (IQR)	Median (IQR)			Median (IQR)	Median (IQR)		
6MWT												
Baseline	0 (0)	0 (0)	.666	.066								
Post intervention	30 (212)	0 (0)	.200	.334	30 (135)	0 (103)	.292	.273				
Follow-up	125 (220)	30 (120)	.074	.500	105 (220)	30 (118)	.147	.401	30 (123)	0 (42)	.399	.231
mRS†												
Baseline	4 (1)	5 (1)	.130	.380								
Post intervention	4 (1)	4 (1)	.208	.329	−1 (1)	−1 (1)	.992	.003				
Follow-up	3 (2)	4 (1)	.310	.273	−1 (1)	−1 (2)	.984	.005	0 (1)	0 (1)	.918	.026
BI												
Baseline	12 (4)	11 (5)	.640	.125								
Post intervention	19 (11)	14 (9)	.154	.401	6 (6)	3 (5)	.116	.445				
Follow-up	24 (11)	20 (11)	.139	.418	9 (11)	8 (9)	.292	.294	2 (4)	3 (5)	.970	.010
Inpatient care												
Enrollment to discharge (d)	22 (12)	24 (9)	.264	.313								

NOTE. Between-group comparisons were calculated using the Mann-Whitney *U* test.

∗ Mann-Whitney *U* test.

† mRS: higher values indicate more severe degree of disability or dependence.

### Secondary outcome measures

The change in disability degree (mRS) from baseline to follow-up was similar in the intervention and control groups (median, −1 [IQR, 1] vs –1, [IQR, 2], respectively; *P*=.984, *d*=0.005) (see table 2). The change in BI from baseline to follow-up was similar in the intervention and control groups (median, 9 [IQR, 11] vs 8, [IQR, 9], respectively; *P*=.292, *d*=0.294). The number of inpatient care days from stroke enrollment to discharge was similar in the intervention and control groups (median, 22d [IQR, 12d] vs 24d [IQR, 9d], respectively; *P*=.264, *d*=0.313).

### Subgroup analysis, participants with NIHSS 7-12 and NIHSS 13-42

#### Primary outcome measure

**Fig 2:**
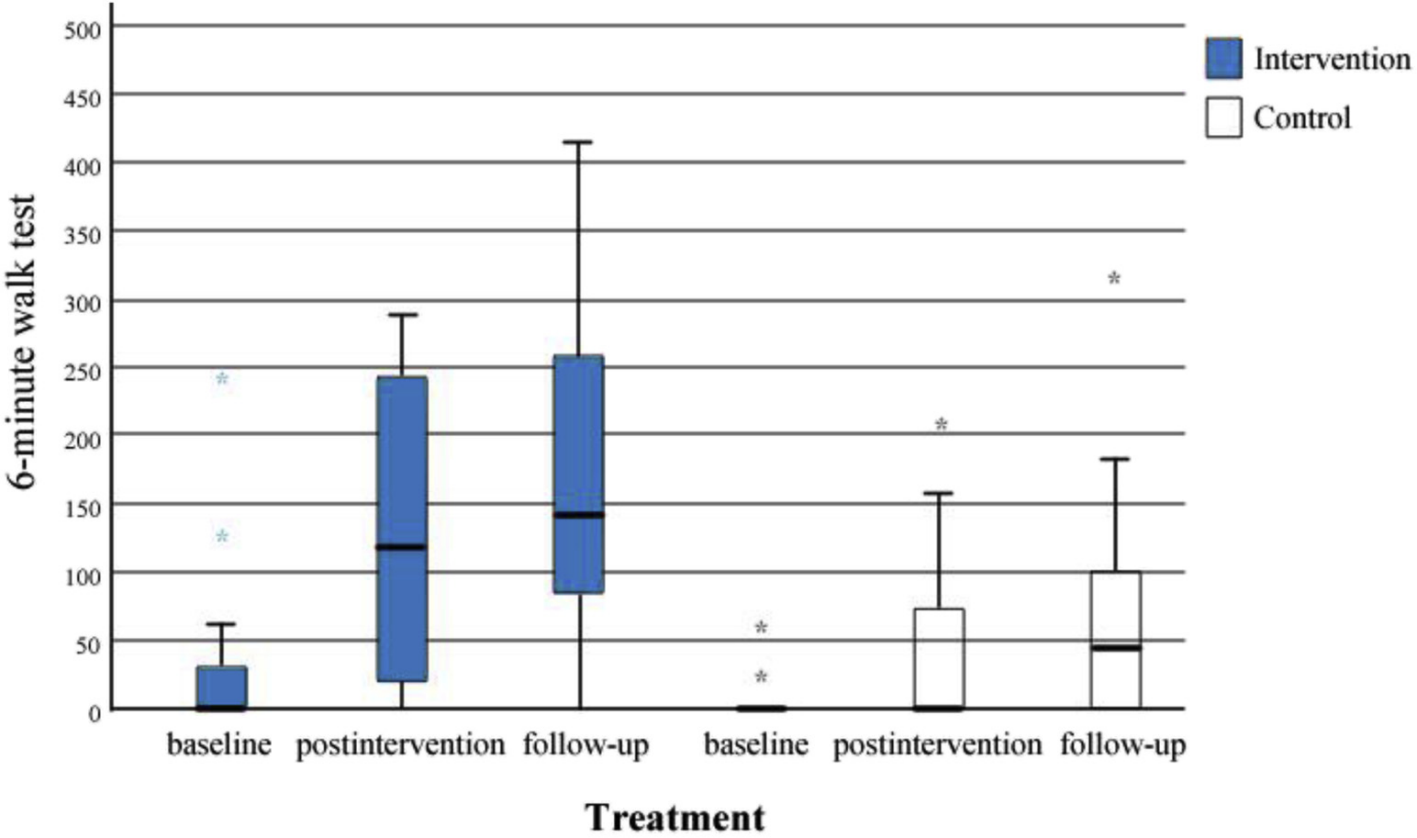
Six-minute walk test measured at baseline, at post intervention, and at follow-up in patients with NIHSS 7-12.

In participants with NIHSS 7-12, the change in walking distance (6MWT) from baseline to follow-up was numerically larger in the intervention group than in the control group, but the difference was not statistically significant: (median [IQR]=113m [212m] vs 30m [116m], respectively; *P*=.083, *d*=.718) (table 3 and fig 2).

**Table 3:** Primary and secondary outcome measures in patients with NIHSS 7-12, comparisons between groups and over time

Measures	Intervention NIHSS 7-12 (n=12)	Control NIHSS 7-12 (n=15)	*P* Value∗	*d* Effect Size	Change From Baseline				Change From Post Intervention			
					Intervention NIHSS 7-12 (n=12)	Control NIHSS 7-12 (n=15)	*P* Value∗	*d* Effect Size	Intervention NIHSS 7-12 (n=12)	Control NIHSS 7-12 (n=15)	*P* Value∗	*d* Effect Size
	Median (IQR)	Median (IQR)			Median (IQR)	Median (IQR)			Median (IQR)	Median (IQR)		
6MWT												
Baseline	0 (45)	0 (0)	.548	.246								
Post intervention	120 (242)	0 (80)	.028	.920	74 (170)	0 (80)	.059	.787				
Follow-up	143 (230)	45 (116)	.014	1.064	113 (213)	30 (116)	.083	.718	53 (177)	0 (51)	.516	.256
mRS†												
Baseline	4 (1)	4 (1)	.067	.752								
Post intervention	3 (1)	4 (1)	.025	.945	−1 (1)	−1 (1)	.456	.304				
Follow-up	3 (1)	3 (2)	.236	.473	−1 (2)	−1 (2)	.867	.066	0 (1)	0 (1)	.456	.304
BI												
Baseline	14 (8)	13 (5)	.139	.598								
Post intervention	22 (7)	15 (8)	.075	.730	8 (6)	5 (6)	.167	.556				
Follow-up	27 (9)	20 (10)	.053	.799	9 (10)	9 (9)	.516	.256	2 (4)	3 (3)	.905	.047
Inpatient care												
Enrollment to discharge (d)	18 (11)	25 (11)	.053	.799								

NOTE. Between-group comparisons were calculated using the Mann-Whitney *U* test.

∗ Mann-Whitney *U* test.

† mRS: higher values indicate more severe degree of disability or dependence.

In participants with NIHSS 13-42, the change in walking distance (6MWT) was numerically higher in the intervention group than in the control group, but the difference was not statistically significant (median, 30m [IQR, 220] vs 15m [IQR, 158m], respectively; *P*=.767, *d*=0.121) (table 4).

**Table 4:** Primary and secondary outcome measures in patients with NIHSS 13-42, comparisons between groups and over time

Measures	Intervention NIHSS 13-42 (n=11)	Control NIHSS 13-42 (n=14)	*P* Value∗	*d* Effect Size	Change From Baseline				Change From Post Intervention			
					Intervention NIHSS 13-42 (n=11)	Control NIHSS 13-42 (n=14)	*P* Value∗	*d* Effect Size	Intervention NIHSS 13-42 (n=11)	Control NIHSS 13-42 (n=14)	*P* Value∗	*d* Effect Size
	Median (IQR)	Median (IQR)			Median (IQR)	Median (IQR)			Median (IQR)	Median (IQR)		
6MWT												
Baseline	0 (0)	0 (0)	.767	.121								
Post intervention	0 (135)	0 (176)	.767	.132	0 (135)	0 (150)	.809	.110				
Follow-up	30 (220)	15 (158)	.767	.121	30 (220)	15 (158)	.767	.121	0 (125)	0 (56)	.609	.220
mRS†												
Baseline	5 (1)	5 (0)	.809	.099								
Post intervention	4 (1)	4 (1)	.572	.243	−1 (1)	−1 (1)	.467	.310				
Follow-up	4 (1)	4 (2)	.767	.121	−1 (1)	−1 (2)	.851	.088	-1 (1)	0 (1)	.373	.367
BI												
Baseline	10 (1)	10 (2)	.609	.209								
Post intervention	14 (8)	14 (7)	.893	.055	4 (6)	3 (4)	.572	.231				
Follow-up	18 (12)	19 (11)	.851	.088	8 (12)	8 (10)	.434	.322	2 (3)	3 (6)	.979	.011
Inpatient care												
Enrollment to discharge (d)	27 (8)	24 (9)	.647	.198								

NOTE. Between-group comparisons were calculated using the Mann-Whitney *U* test.

∗ Mann-Whitney *U* test.

† mRS: higher values indicate more severe degree of disability or dependence.

#### Secondary outcome measures

In participants with NIHSS 7-12, the change in disability degree (mRS) from baseline to follow-up was similar in the intervention and control groups (median, −1 [IQR, 2] vs −1 [IQR, 2], respectively; *P*=.867, *d*=0.066) (see table 3).

In participants with NIHSS 13-42, the change in disability degree (mRS) from baseline to follow-up was similar in the 2 groups (median, −1 [IQR, 1] vs −1 [IQR, 2], respectively; *P*=.851, *d*=0.121) (see table 4).

In participants with NIHSS 7-12, the change in BI from baseline to follow-up was similar in the 2 groups (median, 9 [IQR, 10] vs 9 [IQR, 9], respectively; *P*=.516, *d*=0.256) (see table 3).

In participants with NIHSS 13-42, the change in BI from baseline to follow-up was similar in the 2 groups (median, 8 [IQR, 12] vs 8 [IQR, 10], respectively; *P*=.434, *d*=0.322) (see table 4).

In participants with NIHSS 7-12, the number of inpatient care days from stroke enrollment to discharge was numerically lower in the intervention group than in the control group, but the difference was not statistically significant (median, 18d [IQR, 11d] vs 25d [IQR, 11d], respectively: *P*=.053, *d*=0.799) (see table 3).

In participants with NIHSS 13-42, the number of inpatient care days from stroke enrollment to discharge was similar in the intervention and control groups (median, 27d [IQR, 8d] vs 24d [IQR, 9d], respectively: *P*=.647, *d*=0.198) (see table 4).

## Discussion

In this dual-center randomized controlled study, which may have been underpowered, early exercise after stroke was not superior to standard inpatient rehabilitation in improving 6MWT, mRS, BI, and inpatient care days. However, in the subgroup with NIHSS 7-12 there was a trend of borderline statistical significance toward benefit for the intervention group regarding median change in 6MWT and mRS and in inpatient care days. In those with more severe stroke there were no significant differences between the intervention and control groups. Relatively few trials^27^, ^28^, ^29^, ^30^, ^31^ have started early rehabilitation within this acute poststroke phase, which has been limited to days 1-7 by the Stroke and Rehabilitation Roundtable Taskforce.^1^ This time perspective, which was used in the current study, represents an important treatment target to maximize the potential of restorative interventions but limits the number of comparable studies. To our knowledge our study is the only study that has used in-bed cycle exercise in the acute phase after stroke. Although the study showed no significant difference between in-bed cycle exercise and usual care alone, it is noteworthy that it was feasible and safe to carry out exercise in participants with severe stroke in the acute phase. No deaths occurred during the study or during follow-up.

There are some concerns about potential harm of early mobilization, particularly in the first 24 hours after stroke onset.^32^ These concerns include hemodynamic considerations, such as fears that raising the patient’s head early after stroke will impair cerebral blood flow and cerebral perfusion. Marzolini et al^33^ concluded that mobilization strategies in early phases post stroke need to mitigate the risk associated with orthostatic hypotension and extended blood pressure elevation as well as the potential for postexercise hypotension. In-bed cycle exercise as used in the current study could be a way to stimulate cerebral repair processes to reach higher functional outcomes without affecting blood pressure adversely.

### Study limitations

This is one of the first randomized controlled trials to investigate the effect of in-bed cycle exercise in the acute phase after stroke. The longitudinal design allowed us to study changes in effects over time. The participants were included from 2 regional clinics, and the results may be generalizable to similar hospital settings. The study does, however, have limitations. First, a larger than expected variability in the outcome measures may have contributed to a lack of statistical power. This calls for a cautious interpretation of the neutral study result. In particular, the subgroup findings should be considered as hypothesis generating. Larger studies are needed to confirm our results*.* Second, time from onset to baseline and post intervention is presented in days and could have been more precisely specified in hours. Third, the intervention period of 3 weeks may have been too short to show additional benefits compared with the control group. Fourth, this study did not gather any information about each patient’s activity levels during and after the intervention. Fifth, the use of in-bed cycle ergometry can be questioned regarding improving walking ability because it is not a walking-specific intervention. However, in-bed cycle ergometry was one of the few possible exercise interventions in this group of participants. Finally, the assessment in this trial was not blinded.

## Conclusions

Although this study may have been underpowered, we found that early in-bed cycle exercise did not favorably influence outcome after 3 months with respect to walking ability, degree of disability, and inpatient care days in participants with stroke NIHSS 7-42. However, there was a trend of borderline statistical significance toward benefit in the subgroup of participants with NIHSS 7-12, in which the intervention group improved more in walking ability and degree of disability and needed fewer inpatient care days than the control group. Future studies should examine whether certain groups of participants benefit from early in-bed cycle exercise in the acute phase after stroke. A larger study, or pooled data from smaller studies, is needed to confirm our results.

## Acknowledgments

We thank the staff at the Stroke Department Vrinnevi Hospital, Norrköping, and Stroke Department Höglandssjukhuset, Eksjö, for inclusion and testing of participants. We also thank Erwin E. Schmitz, MD, for help with data processing.

## Suppliers

a. MOTOmed Letto2; RECK-Technik GmbH & Co KG.
b. SPSS version 25; IBM.
